# Impact of chronic endometritis on assisted reproductive technology outcomes: a propensity score inverse probability weighting cohort study

**DOI:** 10.3389/fcell.2026.1749173

**Published:** 2026-01-28

**Authors:** Wenjie Huang, Liqiong Duan, Liuyan Wei, Ni Tang, Jia Wei, Xuechang Chou, Yaping Ma, Lili Wei, Ming Zhang, Fangmei Lu, Li Fan, Kai Wang, Huawei Wang

**Affiliations:** 1 Reproductive Genetics Department / Obstetrics Department, The First Affiliated Hospital of Kunming Medical University, Kunming, Yunnan, China; 2 Department of Reproductive Medicine, Guangzhou Women and Children’s Medical Center Liuzhou Hospital, Liuzhou, Guangxi, China; 3 Liuzhou Maternity and Child Healthcare Hospital, Liuzhou, Guangxi, China

**Keywords:** assisted reproductive technology, CD138, chronic endometritis, embryo transfer, pregnancy outcome

## Abstract

**Background:**

Chronic endometritis (CE), characterized by CD138^+^ plasma cell infiltration, has been proposed to impair reproductive outcomes in assisted reproductive technology (ART). However, current evidence remains inconsistent, and diagnostic criteria vary widely. This study aimed to evaluate whether CE diagnosis and antibiotic treatment influence clinical pregnancy, live birth, and miscarriage outcomes following *in vitro* fertilization with fresh embryo transfer (IVF-ET) and frozen embryo transfer (FET).

**Methods:**

We retrospectively analyzed 3,041 embryo transfer cycles (1,507 IVF-ET; 1,534 FET) from 1,401 infertile women treated at the Reproductive Medicine Center of Liuzhou Hospital, Guangzhou Women and Children’s Medical Center (2008–2023). Chronic endometritis (CE) was primarily defined as the presence of ≥1 CD138^+^ plasma cell per 10 high-power fields (HPFs, ×400). Standard therapy was doxycycline 100 mg twice daily for 14 days. Outcomes were analyzed per transfer cycle using Poisson regression with robust standard errors and propensity score weighting.

**Results:**

Untreated CE patients had comparable live birth and clinical pregnancy rates to non-CE patients in both IVF-ET (live birth 37.7% vs. 36.7%) and FET (39.0% vs. 37.9%), and were also associated with a lower observed miscarriage risk after IVF-ET (adjusted RR 0.67, 95% CI 0.46–0.97). Among CE patients, antibiotic treatment did not improve outcomes compared with no treatment (IVF-ET live birth 36.8% vs. 37.7%; FET 41.5% vs. 38.7%; all p > 0.05). Similarly, post-treatment “cured” and “persistent” CE groups showed no significant differences in live birth or miscarriage rates in either transfer type. Exploratory analyses revealed context-dependent trends, but most interactions were nonsignificant.

**Conclusion:**

In this large single-center cohort, CE defined by CD138^+^ plasma cell infiltration was not associated with reduced clinical pregnancy or live birth rates, and no statistically significant benefit of antibiotic treatment was observed. The observed reduction in miscarriage risk among CE patients suggests complex immunological dynamics. Routine CE screening in all ART candidates may be unnecessary, and targeted evaluation for high-risk subgroups warrants further investigation.

## Introduction

Chronic endometritis (CE) is a persistent inflammation of the endometrium, typically caused by microbial infection or immune dysregulation, and is histologically characterized by plasma cell infiltration within the endometrial stroma (Moreno et al., 2018). Epidemiological studies indicate that CE is relatively common among women of reproductive age, particularly in infertile populations, with reported prevalence rates of 56% in recurrent spontaneous abortion (RSA), 57.5% in recurrent implantation failure (RIF), and 51.7% in unexplained infertility (UI) (McQueen et al., 2015; Cicinelli et al., 2015; Gu et al., 2023). The current gold standard for CE diagnosis is histopathological identification of plasma cells in the endometrium, usually detected by Syndecan-1 (CD138) immunohistochemical staining (Klimaszyk et al., 2023). However, diagnostic criteria vary considerably, with some defining CE as the presence of 1–5 plasma cells per high-power field (HPF) and others requiring ≥1 plasma cell across 10 HPFs at ×400 magnification (Bouet et al., 2016; Pirtea et al., 2021; Liu et al., 2018), leading to substantial heterogeneity (Margulies et al., 2021).

Clinical evidence suggests that CE may impair reproductive outcomes. Several studies have reported lower clinical pregnancy rates in CE patients compared with non-CE patients (Chen et al., 2021). Furthermore, antibiotic treatment and subsequent cure have been associated with improved IVF outcomes, yielding results comparable to those of non-CE women (Vitagliano et al., 2022). A systematic review and meta-analysis further demonstrated that antibiotic therapy may improve IVF outcomes in RIF patients, but emphasized the importance of confirming CE cure prior to embryo transfer (Vitagliano et al., 2018).

Given its potential association with adverse reproductive outcomes, some investigators advocate routine CE screening in ART candidates, suggesting that timely diagnosis and treatment may improve outcomes. However, CE diagnosis remains controversial, largely due to the absence of standardized thresholds, which may cause misclassification. Accordingly, the American Society for Reproductive Medicine does not recommend routine endometrial biopsy before ART, citing limited sensitivity and specificity (Cicinelli et al., 2022). Moreover, reported associations between CE and ART outcomes are inconsistent: while some studies show no significant relationship with implantation, pregnancy, or live birth rates (Kasius et al., 2011; Herlihy et al., 2022), others suggest apparent benefits of antibiotic therapy that may reflect regression to the mean rather than true treatment effects (Cicinelli et al., 2022). These discrepancies likely stem from variations in diagnostic criteria, study populations, and treatment protocols.

Therefore, this study aimed to systematically evaluate the impact of CE diagnosis and treatment on ART outcomes by comparing (i) non-CE patients versus untreated CE patients, (ii) treated versus untreated CE patients, and (iii) cured versus persistent CE patients. This analysis was designed to provide robust evidence to inform clinical decision-making on routine CE screening and management.

## Materials and methods

### Study design

This retrospective cohort study was conducted at the Reproductive Medicine Center of Liuzhou Hospital, Guangzhou Women and Children’s Medical Center. The study protocol was approved by the institutional ethics committee (No. 2025-147). Owing to the retrospective design and absence of identifiable patient information, informed consent was waived.

### Study population

We included infertile women who underwent autologous IVF-ET or FET cycles between August 2008 and December 2023. All patients underwent hysteroscopy with concurrent endometrial biopsy and CD138 immunohistochemical staining due to a history of embryo transfer failure, recurrent pregnancy loss (RPL), abnormal uterine bleeding (AUB), or suspected endometrial abnormalities on ultrasound (e.g., polyps, adhesions).

Chronic endometritis (CE) was diagnosed based on histopathological evaluation of endometrial biopsy specimens with CD138 immunohistochemistry. The predefined diagnostic criterion for CE was the presence of ≥1 CD138^+^ plasma cell per 10 high-power fields (HPFs, ×400) (Cicinelli et al., 2019). During the earlier study period (2008–2019), pathology reports were predominantly qualitative and documented the presence or absence of CD138^+^ plasma cells without providing numerical counts; reports indicating CD138^+^ plasma cell positivity were therefore considered diagnostically equivalent to meeting this predefined threshold, whereas reports explicitly stating absence of CD138^+^ plasma cells were classified as non-CE. In the later study period (2020–2023), pathology reports routinely provided quantitative plasma cell counts across HPFs, allowing direct application of the same diagnostic criterion. Accordingly, CE classification for the full cohort was based on this unified threshold, while stratification by plasma cell density (0, 1–4, and ≥5 cells/HPF) was performed only as a sensitivity analysis in cycles with quantitative reporting.

Treatment strategies for CE patients were determined by the attending physician in consultation with the patient. Standard antibiotic therapy consisted of doxycycline 100 mg orally twice daily for 14 days. For patients allergic to doxycycline, levofloxacin 500 mg once daily combined with metronidazole 200 mg three times daily for 14 days was prescribed (Zhang et al., 2024). Given the uncertainty regarding the clinical significance of mild CE, some patients did not receive antibiotic treatment as part of routine clinical practice. Exclusion criteria included cycles involving donor oocytes, donor sperm, or thawed oocytes; cycles with preimplantation genetic testing (PGT); antibiotic use within 3 months before biopsy; autoimmune disease; adenomyosis; and uterine malformations. We included only patients who underwent standardized post-treatment reassessment for the analysis of treatment outcomes. Although most patients received follow-up evaluations, some declined based on individual preferences and clinical circumstances, resulting in a smaller reassessed group. This may limit the generalizability of our findings, but including only those with reassessment ensured consistency in diagnostic standards.

Baseline demographic and clinical information were retrieved from medical records, including female age (verified from identification documents), duration of infertility (self-reported), infertility diagnosis, ovarian stimulation protocol, and fertilization method. Height and weight were measured at the first visit to calculate BMI. Embryo-related information (number and quality of transferred embryos) was recorded by the embryology laboratory, and serum hormone levels were obtained from laboratory databases.

### Ovarian stimulation protocols

All patients received individualized ovarian stimulation based on physician assessment. Commonly used protocols included GnRH antagonist and agonist regimens. Gonadotropins were administered subcutaneously to stimulate follicular development, and follicle growth was monitored by transvaginal ultrasound and serum estradiol, luteinizing hormone, and progesterone levels. Final oocyte maturation was triggered with human chorionic gonadotropin (hCG) and/or a GnRH agonist once follicles reached maturity. Dosage and drug combinations followed established protocols (Huang et al., 2025), with adjustments according to patient characteristics and ovarian response.

### Fertilization and embryo culture

Fertilization was performed by conventional IVF or ICSI as clinically indicated. For IVF, approximately 50,000 motile spermatozoa were co-incubated with each oocyte for 4–6 h. After cumulus cell removal, normally fertilized oocytes were transferred into fresh culture medium. For ICSI, morphologically normal spermatozoa were immobilized and injected into mature (MII) oocytes. Embryos were cultured under standard laboratory conditions, and fertilization was assessed 16–18 h after insemination.

### Embryo transfer protocols

Embryo transfer was performed either in fresh cycles or FET cycles. In fresh cycles, embryos were transferred on day 3 or at the blastocyst stage (day 5–6), depending on embryo quality. FET cycles were performed at least 2 months after the fresh cycle, using natural, clomiphene-induced, or hormone replacement regimens for endometrial preparation. Embryos with a post-thaw survival rate >50% were considered suitable for transfer. A maximum of two embryos were transferred per FET cycle.

### Outcomes

The primary outcomes was live birth. Secondary outcomes included clinical pregnancy and miscarriage. Clinical pregnancy was defined as the ultrasonographic detection of an intrauterine gestational sac, or in the absence of imaging, clinical documentation of live birth, ectopic pregnancy, or miscarriage. Live birth was defined as delivery of one or more live infants. Miscarriage was defined as spontaneous loss of an intrauterine pregnancy before 20 weeks of gestation.

### Covariates

Baseline covariates included female and male clinical and laboratory variables previously reported to influence ART outcomes.

For fresh embryo transfer cycles, covariates included female age, BMI, AMH level, gravidity, parity, duration of infertility, baseline estradiol (E2), follicle-stimulating hormone (FSH), luteinizing hormone (LH), total gonadotropin dose, endometrial thickness on HCG day, number of embryos transferred, fertilization method (IVF or ICSI), day of transfer (day 3 or day 5/6), type of infertility (primary or secondary), infertility cause (tubal, male, ovulatory, endometriosis, or other), and ovarian stimulation protocol (agonist, antagonist, mild stimulation, or natural cycle).

For FET cycles, covariates included female age, BMI, AMH, gravidity, parity, duration of infertility, endometrial thickness on HCG day, number of embryos transferred, fertilization method, type of infertility, day of transfer, and endometrial preparation regimen (natural or programmed).

### Statistical analysis

Analyses were performed per transfer cycle, accounting for potential correlation among repeated cycles from the same patient by using cluster-robust standard errors (patient-level clustering). Statistical analyses were performed using SPSS version 26.0 and R (RStudio), with two-sided significance set at 0.05. Outcome variables had no missing data. Missingness for other variables was <5% and handled by single imputation, using the median for continuous variables and the most frequent category (mode) for categorical variables.

To reduce confounding, propensity score (PS) weighting with stabilized inverse probability weighting (IPW) was applied. PS were estimated using logistic regression models including all baseline covariates (17 for fresh cycles and 12 for FET cycles). Model performance was assessed using the c-statistic and standardized mean differences (SMD), with SMD <0.1 indicating adequate balance.

Weighted descriptive statistics were used. For clinical outcomes (clinical pregnancy, live birth, miscarriage), Poisson regression models with robust standard errors were fitted: (Moreno et al., 2018): unadjusted model; (McQueen et al., 2015); multivariable-adjusted model including all baseline covariates; (Cicinelli et al., 2015); PS-weighted model; and (Gu et al., 2023) PS-weighted model with additional covariate adjustment. Results were reported as relative risks (RRs) with 95% confidence intervals (CIs).

To assess potential effect modification, interaction analyses were performed within the PS-weighted framework. Interaction terms between treatment group and baseline covariates were entered into Poisson regression models, and significant interactions were reported with corresponding RRs and 95% CIs.

## Results

### Study population

A total of 1,401 women met the inclusion criteria, contributing 3,041 IVF/ICSI transfer cycles (1,507 IVF-ET and 1,534 FET). For the comparison between untreated CE and non-CE patients, 966 IVF-ET cycles (746 first and 220 subsequent) and 1,014 FET cycles (499 first and 515 subsequent) were analyzed. Among CE patients, 1,075 IVF-ET cycles (787 first and 288 subsequent) and 1,059 FET cycles (533 first and 526 subsequent) were included for treated versus untreated comparisons. Following treatment, patients were further divided into persistent CE and cured CE groups, comprising 284 IVF-ET cycles (205 first and 79 subsequent) and 282 FET cycles (132 first and 150 subsequent).

Overall, 72%–77% of transfers were IVF and 23%–28% were ICSI. Approximately 60% of embryos were transferred at the blastocyst stage (day 5–6), with the remainder at the cleavage stage (day 3). In FET cycles, >70% used programmed endometrial preparation, while in IVF-ET cycles GnRH agonist and antagonist regimens were most common.

### Propensity score model

Baseline characteristics are shown in Table 1 and Supplementary Tables S1–S2 for IVF-ET cycles, and Supplementary Tables S3–S5 for FET cycles. Before weighting, significant imbalances were observed between non-CE and untreated CE groups in gravidity, parity, duration of infertility, gonadotropin dose, and endometrial thickness (Table 1). Among CE patients, infertility diagnosis differed between treated and untreated groups (Supplementary Table S1), while persistent versus cured CE groups differed in AMH, gravidity, infertility duration, baseline LH, and gonadotropin dose (Supplementary Table S2). After PS IPW, all SMDs were <0.1, confirming satisfactory covariate balance.

**TABLE 1:** Baseline characteristics of non-treated women with Non-CE and CE undergoing fresh IVF cycles before and after propensity score inverse probability weighting (PS IPW).

Baseline characteristic	Overall, n = 966	Unweighted		SMD	PS weighted		SMD
		Non-CE, n = 432	CE, n = 534		Non-CE, n = 432.35	CE, n = 533.83	
Age	35.15 (4.80)	34.94 (4.73)	35.32 (4.85)	0.078	35.11 (4.65)	35.11 (4.90)	0.001
BMI (kg/m2)	22.29 (3.21)	22.26 (3.13)	22.32 (3.27)	0.019	22.24 (3.08)	22.26 (3.27)	0.007
AMH (ng/mL)	2.52 (1.99)	2.58 (2.20)	2.46 (1.80)	0.060	2.50 (2.05)	2.51 (1.91)	0.001
Gravidity	1.56 (1.61)	1.66 (1.71)	1.48 (1.51)	0.112	1.55 (1.62)	1.55 (1.57)	0.003
Parity	0.36 (0.58)	0.31 (0.52)	0.39 (0.62)	0.134	0.35 (0.55)	0.35 (0.59)	0.003
Infertility duration (y)	5.12 (4.37)	4.81 (4.18)	5.37 (4.51)	0.128	5.21 (4.46)	5.16 (4.36)	0.010
Basal E2 (pg/mL)	51.96 (96.74)	52.68 (84.74)	51.37 (105.53)	0.014	52.39 (81.11)	51.99 (113.80)	0.004
Basal FSH (mIU/mL)	6.34 (3.23)	6.50 (3.11)	6.21 (3.32)	0.091	6.38 (2.98)	6.40 (3.70)	0.007
Basal LH (mIU/mL)	3.73 (3.74)	3.69 (3.89)	3.76 (3.61)	0.018	3.73 (4.18)	3.75 (3.29)	0.005
Total Gn	1923.76 (817.44)	1860.17 (834.84)	1975.20 (800.18)	0.141	1925.01 (838.27)	1920.72 (813.00)	0.005
Endometrial thickness	11.01 (2.42)	10.67 (2.28)	11.28 (2.49)	0.254	10.98 (2.34)	11.00 (2.44)	0.007
No. of embryos transferred	1.57 (0.50)	1.56 (0.50)	1.57 (0.49)	0.030	1.57 (0.50)	1.57 (0.50)	0.003
Fertilization method (%)	​	​	​	0.043	​	​	0.011
IVF	737 (76.3)	334 (77.3)	403 (75.5)	​	327.0 (75.6)	406.2 (76.1)	​
ICSI	229 (23.7)	98 (22.7)	131 (24.5)	​	105.3 (24.4)	127.6 (23.9)	​
Day of transfer (%)	​	​	​	0.081	​	​	0.002
Day 3	726 (75.2)	333 (77.1)	393 (73.6)	​	323.2 (74.8)	399.4 (74.8)	​
Day 5/6	240 (24.8)	99 (22.9)	141 (26.4)	​	109.1 (25.2)	134.4 (25.2)	​
Type of infertility (%)	​	​	​	0.026	​	​	0.002
Primary	324 (33.5)	142 (32.9)	182 (34.1)	​	146.1 (33.8)	179.7 (33.7)	​
Secondary	642 (66.5)	290 (67.1)	352 (65.9)	​	286.3 (66.2)	354.1 (66.3)	​
Infertility diagnosis (%)	​	​	​	0.111	​	​	0.025
Tubal factor	624 (64.6)	275 (63.7)	349 (65.4)	​	277.1 (64.1)	344.7 (64.6)	​
Male factor	166 (17.2)	74 (17.1)	92 (17.2)	​	77.3 (17.9)	93.8 (17.6)	​
Ovulatory	100 (10.4)	52 (12.0)	48 (9.0)	​	42.7 (9.9)	53.0 (9.9)	​
Endometriosis	22 (2.3)	8 (1.9)	14 (2.6)	​	11.5 (2.7)	12.5 (2.3)	​
Other	54 (5.6)	23 (5.3)	31 (5.8)	​	23.7 (5.5)	29.8 (5.6)	​
Ovarian stimulation protocol, No. (%)		​	​	0.167	​	​	0.024
Agonist	541 (56.0)	228 (52.8)	313 (58.6)	​	240.4 (55.6)	298.2 (55.9)	​
Antagonist	386 (40.0)	180 (41.7)	206 (38.6)	​	174.4 (40.3)	213.1 (39.9)	​
Mild stimulation	35 (3.6)	21 (4.9)	14 (2.6)	​	15.9 (3.7)	21.0 (3.9)	​
Natural cycles	4 (0.4)	3 (0.7)	1 (0.2)	​	1.7 (0.4)	1.5 (0.3)	​

Abbreviations: BMI, body mass index (calculated as weight in kilograms divided by height in meters squared); AMH, anti-Müllerian hormone; E2, estradiol; FSH, follicle-stimulating hormone; LH, luteinizing hormone; Gn, gonadotropin; SMD, standardized mean difference; IVF, *in vitro* fertilization; ICSI, intracytoplasmic sperm injection.

^a^ Values are presented as mean ± standard deviation for continuous variables and as number (percentage) for categorical variables.

^b^ SMD (standardized mean difference) was used to assess the balance of covariates between Non-CE, and CE, groups, both before and after PS IPW.

^c^ Gravidity refers to the number of times a woman has been pregnant, regardless of the outcome; parity refers to the number of pregnancies carried to viable gestational age.

^d^ Infertility diagnosis categories are not mutually exclusive; multiple causes may be assigned to a single patient. “Tubal factor” refers to occlusion or damage of the fallopian tubes. “Male factor” refers to abnormalities in semen quality or function. “Ovulatory” refers to disorders affecting ovulation. “Endometriosis” includes women with diagnosed endometrial-like lesions outside the uterus. “Other” includes rare causes such as uterine anomalies or unexplained infertility.

^e^ Fertilization method includes conventional IVF, and ICSI. ICSI, is typically used in cases of male factor infertility or previous fertilization failure.

^f^ The day of embryo transfer is defined as either cleavage-stage embryo transfer (day 3) or blastocyst-stage embryo transfer (day 5/6), depending on embryo development.

^g^ Ovarian stimulation protocols include GnRH, agonist and antagonist regimens, mild stimulation protocols, and natural cycles.

^h^ Percentages may not total to 100% due to rounding.

^i^ Sample size after propensity score weighting is presented with decimal values because inverse probability weighting generates weighted pseudo-populations rather than integer counts.

In FET cycles, non-CE and CE groups differed in age, AMH, parity, endometrial thickness, number of embryos transferred, and infertility type (Supplementary Table S3). Treated versus untreated CE groups differed in parity (Supplementary Table S4), and persistent versus cured CE groups differed in age, AMH, gravidity, parity, infertility duration, embryo transfer day, and fertilization method (Supplementary Table S5). All imbalances were resolved after weighting (SMD <0.1).

### Pregnancy outcomes

As shown in Table 2, pregnancy outcomes were comparable between untreated CE and non-CE patients. After fresh embryo transfer (IVF-ET), the live birth rate was 37.7% vs. 36.7%, and the clinical pregnancy rate was 46.8% vs. 50.0% in the CE and non-CE groups, respectively. Neither outcome differed significantly (unadjusted RR, 1.06 [95% CI, 0.89–1.26]; adjusted RR [aRR], 1.07 [95% CI, 0.91–1.26]). However, the risk of miscarriage was significantly lower among CE patients (9.1% vs. 13.3%; unadjusted RR, 0.64 [95% CI, 0.45–0.93]; aRR, 0.67 [95% CI, 0.46–0.97]; PS IPW RR, 0.66 [95% CI, 0.46–0.97]). This reduction remained consistent across models.

**TABLE 2:** Pregnancy outcomes in untreated CE vs. Non-CE patients undergoing fresh and FET cycles after PS IPW.

Outcomes	Events, no/total (%)		Relative risk (95% Cl)			
	CE (533.8)	Non-CE (432.4)	Unadjusted	Multivariable adjusted^a^	PS IPW^b^	PS IPW + multivariable adjusted^a^ ^,^ ^b^
Live birth	201.4 (37.7)	158.5 (36.7)	1.06 (0.89–1.26)	1.07 (0.91–1.26)	1.03 (0.87–1.22)	1.03 (0.88–1.21)
Clinical pregnancy	249.9 (46.8)	216.0 (50.0)	0.95 (0.83–1.08)	0.96 (0.84–1.09)	0.93 (0.81–1.07)	0.93 (0.82–1.06)
Miscarriage	48.5 (9.1)	57.6 (13.3)	0.64 (0.45–0.93)	0.67 (0.46–0.97)	0.66 (0.46–0.97)	0.66 (0.46–0.96)
FET	CE (538.4)	Non-CE (475.0)	Unadjusted	Multivariable adjusted^c^	PS IPW^d^	PS IPW + multivariable adjusted^c^ ^,^ ^d^
Live birth	210.2 (39.0)	179.8 (37.9)	1.02 (0.86–1.20)	1.02 (0.87–1.19)	1.03 (0.87–1.22)	1.02 (0.87–1.20)
Clinical pregnancy	264.2 (49.1)	240.6 (50.7)	0.96 (0.84–1.09)	0.96 (0.85–1.09)	0.97 (0.85–1.10)	0.96 (0.85–1.09)
Miscarriage	52.0 (9.7)	59.0 (12.4)	0.78 (0.55–1.09)	0.77 (0.55–1.10)	0.77 (0.55–1.10)	0.78 (0.55–1.10)

Outcomes are presented as number of events/total cases (%). Percentages are presented after PS IPW; weighted totals may be fractional. Unadjusted relative risks (RRs) were estimated using Poisson regression models with a log link function, including CE, status as the sole predictor. Adjusted relative risks (aRRs) were obtained from multivariable Poisson regression models with robust standard errors, adjusting for continuous variables and categorical variables.

^a^ Adjusted for age, BMI, AMH, gravidity, parity, infertility duration, basal E2, FSH, LH, total gonadotropin dose, endometrial thickness, number of embryos transferred, fertilization method, day of transfer, type of infertility, infertility diagnosis, and ovarian stimulation protocol.

^b^ The PS, model included age, BMI, AMH, gravidity, parity, infertility duration, basal E2, FSH, LH, total gonadotropin dose, endometrial thickness, number of embryos transferred, fertilization method, day of transfer, type of infertility, infertility diagnosis, and ovarian stimulation protocol.

^c^ Adjusted for age, BMI, AMH, gravidity, parity, infertility duration, endometrial thickness, number of embryos transferred, fertilization method, day of transfer, type of infertility, and endometrial preparation protocol.

^d^ The PS, model included age, BMI, AMH, gravidity, parity, infertility duration, endometrial thickness, number of embryos transferred, fertilization method, day of transfer, type of infertility, and endometrial preparation protocol.

In FET cycles, live birth and clinical pregnancy rates remained similar between groups (live birth, 39.0% vs. 37.9%; unadjusted RR, 1.02 [95% CI, 0.86–1.20]; aRR, 1.02 [95% CI, 0.87–1.19]), while miscarriage risk was lower among CE patients (9.7% vs. 12.4%; PS IPW RR, 0.77 [95% CI, 0.55–1.10]), but did not reach statistical significance (P > 0.05).

As shown in Table 3, pregnancy outcomes were largely similar between treated and untreated CE patients. In fresh cycles, live birth rates were 36.8% vs. 37.7% (unadjusted RR, 0.96 [95% CI, 0.82–1.13]; aRR, 0.94 [95% CI, 0.81–1.10]). Clinical pregnancy rates were likewise comparable (47.9% vs. 46.7%; unadjusted RR, 1.02 [95% CI, 0.90–1.17]; aRR, 1.00 [95% CI, 0.88–1.14]). Although miscarriage appeared slightly higher in treated patients (10.0% vs. 7.3%), this difference was not statistically significant (unadjusted RR, 1.43 [95% CI, 0.96–2.13]; PS IPW aRR, 1.39 [95% CI, 0.93–2.07]).

**TABLE 3:** Pregnancy outcomes in treated vs. untreated CE patients in fresh and FET cycles: comparison of clinical, live birth, and miscarriage rates after PS IPW.

Outcomes	Events, no/total (%)		Relative risk (95% Cl)			
	Treated (543.1)	Untreated (532.8)	Unadjusted	Multivariable adjusted^a^	PS IPW^b^	PS IPW + multivariable adjusted^a^ ^,^ ^b^
Live birth	199.7 (36.8)	200.9 (37.7)	0.96 (0.82–1.13)	0.94 (0.81–1.10)	0.97 (0.82–1.14)	0.96 (0.83–1.12)
Clinical pregnancy	260.2 (47.9)	248.6 (46.7)	1.02 (0.90–1.17)	1.00 (0.88–1.14)	1.03 (0.90–1.17)	1.02 (0.90–1.16)
Miscarriage	54.2 (10.0)	38.7 (7.3)	1.43 (0.96–2.13)	1.39 (0.94–2.06)	1.39 (0.93–2.08)	1.39 (0.93–2.07)
FET	Treated (520.0)	Untreated (539.2)	Unadjusted	Multivariable adjusted^c^	PS IPW^d^	PS IPW + multivariable adjusted^c^ ^,^ ^d^
Live birth	215.5 (41.5)	208.4 (38.7)	1.07 (0.92–1.25)	1.08 (0.93–1.24)	1.07 (0.92–1.25)	1.07 (0.93–1.24)
Clinical pregnancy	262.8 (50.5)	261.4 (48.5)	1.04 (0.91–1.18)	1.05 (0.93–1.18)	1.04 (0.92–1.18)	1.04 (0.92–1.18)
Miscarriage	46.3 (8.9)	51.0 (9.5)	0.92 (0.62–1.35)	0.93 (0.63–1.36)	0.93 (0.62–1.37)	0.93 (0.63–1.36)

^a^ Adjusted for age, BMI, AMH, gravidity, parity, infertility duration, basal E2, FSH, LH, total gonadotropin dose, endometrial thickness, number of embryos transferred, fertilization method, day of transfer, type of infertility, infertility diagnosis, and ovarian stimulation protocol.

^b^ The PS, model included age, BMI, AMH, gravidity, parity, infertility duration, basal E2, FSH, LH, total gonadotropin dose, endometrial thickness, number of embryos transferred, fertilization method, day of transfer, type of infertility, infertility diagnosis, and ovarian stimulation protocol.

^c^ Adjusted for age, BMI, AMH, gravidity, parity, infertility duration, endometrial thickness, number of embryos transferred, fertilization method, day of transfer, type of infertility, and endometrial preparation protocol.

^d^ The PS, model included age, BMI, AMH, gravidity, parity, infertility duration, endometrial thickness, number of embryos transferred, fertilization method, day of transfer, type of infertility, and endometrial preparation protocol.

In FET cycles, all outcomes—including clinical pregnancy (50.5% vs. 48.5%), live birth (41.5% vs. 38.7%), and miscarriage (8.9% vs. 9.5%)—were statistically similar (PS IPW aRR range, 0.92–1.07; P > 0.05).

As summarized in Table 4, no significant differences in pregnancy outcomes were observed between patients with persistent and cured CE after antibiotic treatment. In fresh cycles, the live birth rate was 38.9% vs. 43.6%, and the clinical pregnancy rate was 50.0% vs. 60.0%, respectively. Although the cured group showed numerically higher rates, these did not reach statistical significance (unadjusted RR, 0.92 [95% CI, 0.69–1.23]; aRR, 0.88 [95% CI, 0.65–1.19]). Miscarriage risk was comparable between groups (11.1% vs. 16.4%; aRR, 0.73 [95% CI, 0.38–1.40]).

**TABLE 4:** Pregnancy outcomes in women with persistent vs. cured CE status after treatment, in fresh and FET cycles after PS IPW.

Outcomes	Events, no/total (%)		Relative risk (95% Cl)			
	Persistent CE (157.4)	Cured CE (126.4)	Unadjusted	Multivariable adjusted^a^	PS IPW^b^	PS IPW + multivariable adjusted^a^ ^,^ ^b^
Live birth	61.2 (38.9)	55.1 (43.6)	0.92 (0.69–1.23)	0.88 (0.65–1.19)	0.88 (0.65–1.19)	0.88 (0.65–1.18)
Clinical pregnancy	78.7 (50.0)	75.8 (60.0)	0.85 (0.68–1.07)	0.84 (0.67–1.05)	0.82 (0.66–1.03)	0.83 (0.67–1.04)
Miscarriage	17.4 (11.1)	20.7 (16.4)	0.69 (0.38–1.26)	0.73 (0.38–1.40)	0.68 (0.36–1.29)	0.72 (0.38–1.36)
FET	Persistent CE (151.8)	Cured CE (128.6)	Unadjusted	Multivariable adjusted^c^	PS IPW^d^	PS IPW + multivariable adjusted^c^ ^,^ ^d^
Live birth	59.7 (39.3)	55.3 (43.0)	0.99 (0.73–1.35)	0.94 (0.69–1.27)	0.92 (0.66–1.28)	0.95 (0.70–1.29)
Clinical pregnancy	73.9 (48.7)	67.3 (52.3)	0.99 (0.76–1.28)	0.96 (0.75–1.23)	0.93 (0.71–1.23)	0.98 (0.76–1.26)
Miscarriage	14.2 (9.4)	12.0 (9.3)	0.96 (0.44–2.07)	1.04 (0.44–2.47)	1.00 (0.43–2.36)	1.06 (0.44–2.54)

^a^ Adjusted for age, BMI, AMH, gravidity, parity, infertility duration, basal E2, FSH, LH, total gonadotropin dose, endometrial thickness, number of embryos transferred, fertilization method, day of transfer, type of infertility, infertility diagnosis, and ovarian stimulation protocol.

^b^ The PS, model included age, BMI, AMH, gravidity, parity, infertility duration, basal E2, FSH, LH, total gonadotropin dose, endometrial thickness, number of embryos transferred, fertilization method, day of transfer, type of infertility, infertility diagnosis, and ovarian stimulation protocol.

^c^ Adjusted for age, BMI, AMH, gravidity, parity, infertility duration, endometrial thickness, number of embryos transferred, fertilization method, day of transfer, type of infertility, and endometrial preparation protocol.

^d^ The PS, model included age, BMI, AMH, gravidity, parity, infertility duration, endometrial thickness, number of embryos transferred, fertilization method, day of transfer, type of infertility, and endometrial preparation protocol.

In FET cycles, the results were similar: live birth (39.3% vs. 43.0%) and clinical pregnancy (48.7% vs. 52.3%) rates did not differ significantly (PS IPW aRR range, 0.93–0.98; P > 0.05), and miscarriage risk remained equivalent (aRR, 1.04 [95% CI, 0.44–2.47]).

To address potential concerns regarding diagnostic consistency, we further performed a sensitivity analysis restricted to cycles with quantitative CD138 immunohistochemical reporting (2020–2023), stratifying patients by plasma cell density (0, 1–4, and ≥5 cells/HPF) (Supplementary Table S8). The results remained consistent with the primary analysis, supporting the robustness of our findings when the analysis was limited to a fully quantitative diagnostic framework.

### Interaction analyses

Exploratory subgroup analyses examined potential interactions between CE status and baseline factors (Supplementary Tables S6–S7). In IVF-ET cycles, untreated CE patients with higher gravidity showed improved clinical pregnancy and live birth rates (RR = 1.11 and 1.14, respectively). Interaction analyses indicated effect modification by embryo stage and number of embryos transferred: the association between CE and miscarriage differed for blastocyst versus cleavage-stage transfer (RR = 2.42) and was attenuated with a greater number of embryos transferred (RR = 0.42) (Supplementary Table S6). However, these interaction effects were based on a limited number of miscarriage events within specific strata and were accompanied by wide confidence intervals, indicating substantial statistical uncertainty.

Among treated patients with persistent CE, higher AMH levels were associated with better clinical pregnancy and live birth rates (RR = 1.07–1.08; P < 0.05). Parity showed a similar trend (RR ≈ 1.51), although estimates were imprecise with wide confidence intervals, reflecting a relatively small number of outcome events in these subgroups. In FET cycles, most interactions were nonsignificant. The only significant finding was in persistent CE patients, where ICSI was associated with a markedly lower miscarriage risk compared with IVF (RR = 0.08; 95% CI, 0.01–0.89). This estimate was driven by a very small number of miscarriage events and should therefore be interpreted with caution as an exploratory finding (Supplementary Table S7).

## Discussion

In this large retrospective study including 1,401 women and 3,041 fresh and frozen transfer cycles, we found that untreated CE patients had comparable clinical pregnancy and live birth rates to non-CE patients, but a lower risk of miscarriage. No statistically significant improvement in reproductive outcomes was observed following antibiotic treatment, and comparisons between persistent and cured CE after treatment also revealed no statistically significant differences, although a nonsignificant trend toward higher clinical pregnancy rates was observed in the cured group. Subgroup analyses suggested potential interactions between CE and selected baseline characteristics, indicating that the impact of CE on ART outcomes may be context-dependent. Overall, our findings suggest that CE exerts a limited effect on reproductive outcomes in the general ART population, though it may hold potential clinical relevance in specific subgroups.

Our results are consistent with earlier studies reporting that low-density CD138+ plasma cell infiltration does not significantly affect implantation, clinical pregnancy, or live birth (Li et al., 2024; Li et al., 2021). Herlihy et al. and Morimune et al. similarly demonstrated that CE with mild infiltration carries limited clinical significance (Herlihy et al., 2022; Morimune et al., 2021), although these studies were constrained by small sample sizes and lack of adequate adjustment for baseline differences. Using propensity score weighting in a large cohort, we confirmed and extended these observations. Conversely, other studies have reported a negative association between CE and reproductive outcomes, particularly among women with recurrent implantation failure or endometriosis (Dang et al., 2024; Qiao et al., 2023; Kuroda et al., 2020). Such discrepancies are likely attributable to population heterogeneity. Notably, we observed a reduced miscarriage rate among CE patients, consistent with the findings of Qiao et al. (2023), suggesting that CE may exert complex immunological effects in certain contexts. However, this unexpected association should be interpreted cautiously, as it may be influenced by clinical selection factors and other unmeasured characteristics rather than reflecting a true protective effect of CE. Moreover, this reduction in miscarriage risk was observed primarily in fresh embryo transfer cycles and was not statistically significant in frozen embryo transfer cycles. Interaction analyses showed largely nonsignificant and unstable subgroup effects with wide confidence intervals, further supporting a context-dependent interpretation. Our interaction analyses also suggested effect modification, whereby blastocyst transfer appeared to amplify, whereas multiple embryo transfer appeared to attenuate, the association between CE and miscarriage, highlighting the potential need for individualized embryo transfer strategies.

Previous studies have linked antibiotic treatment to improved pregnancy outcomes in CE patients (Kato et al., 2022; Yang et al., 2014), although most did not include post-treatment reassessment, raising concerns of evaluation bias. In our cohort, antibiotic therapy did not significantly improve outcomes, aligning with the recent findings of Xu et al. (2025). However, differences in baseline inflammatory status between treated and untreated groups may have confounded these results, suggesting that a lack of significant effect does not entirely exclude treatment benefit. Unlike the meta-analyses of Vitagliano et al. (2022); Vitagliano et al., 2018), we did not detect significant advantages in the cured group compared with persistent CE, though a trend toward higher pregnancy rates was observed in IVF-ET cycles. These findings underscore the importance of post-treatment reassessment and suggest that future prospective studies should compare outcomes in patients with comparable baseline inflammation.

The clinical value of routine CE screening before ART remains debated. Cicinelli et al. advocate universal screening and early treatment, whereas Pirtea et al. argue that current evidence is insufficient to support this approach (Cicinelli et al., 2022). Our findings are more aligned with the latter, as we found no compelling evidence to justify universal screening. Drawing parallels with the use of PGT-A, which is most beneficial in selected high-risk groups rather than in all ART patients (The use of preimplantation genetic, 2024), we propose that CE screening may be more appropriate for high-risk subpopulations, such as those with RIF or recurrent miscarriage, rather than the general infertile population.

From a mechanistic perspective, CE may influence ART outcomes by altering the endometrial immune microenvironment and inflammatory response. CE has been associated with abnormal bacterial proliferation and local immune activation, including decreased natural killer cell numbers (Matteo et al., 2009), increased CD8^+^ T cells (Li et al., 2020), downregulation of proinflammatory cytokines such as IL-11, and upregulation of IGFBP-1. These alterations may disrupt embryo–endometrium signaling and impair implantation and trophoblast invasion (Di Pietro et al., 2013). CE has also been linked to dysregulation of genes involved in proliferation and apoptosis (Ki-67, BCL2, BAX), which may compromise endometrial differentiation, decidualization, angiogenesis, and receptivity, ultimately reducing pregnancy and live birth rates (Kitaya et al., 2016). Aberrant release of inflammatory mediators may further disturb uterine contractility, impairing sperm transport and embryo migration (Pinto et al., 2015). Thus, persistent CE after treatment may perpetuate local immune imbalance, interfering with embryo–endometrial interactions and contributing to suboptimal outcomes, even though this was not statistically significant in our study. Conversely, as Negishi et al. highlighted, a certain degree of inflammatory activation is essential for early pregnancy processes such as implantation and trophoblast invasion, whereas both insufficient and excessive inflammation may lead to adverse outcomes (Negishi and Morita, 2024; Förger and Villiger, 2020; Griffith et al., 2017) This dual role may explain why untreated CE patients had similar pregnancy and live birth rates to non-CE patients, but paradoxically exhibited lower miscarriage rates.

This study represents one of the largest investigations to date in this field, including both IVF-ET and FET cycles. By incorporating multiple baseline characteristics and applying PS IPW to achieve covariate balance, we minimized residual confounding and enhanced the validity of comparisons. The analysis extended beyond untreated versus non-CE patients to include treated versus untreated and persistent versus cured CE, thereby offering greater clinical relevance. Moreover, the use of real-world clinical data improves the external validity of our findings. Nonetheless, several limitations should be acknowledged. First, as a retrospective observational study, causality cannot be inferred. Second, the single-center design may limit generalizability. Third, although PS IPW addressed many confounders, unmeasured factors such as genetic background and environmental influences could not be excluded. The long study period also introduced potential diagnostic heterogeneity, as earlier pathology reports were qualitative whereas later cases adopted standardized quantitative definitions, and IVF laboratory techniques and clinical practice (including embryo quality assessment and transfer strategies) evolved substantially over time. In addition, the relatively small sample sizes in the treated and post-treatment subgroups may have limited the statistical power to detect small or moderate benefits of antibiotic therapy. Finally, baseline inflammatory status may have differed between treated and untreated CE patients, possibly underestimating treatment effects. In addition, given the multiple subgroup and interaction analyses performed, the risk of false-positive findings cannot be excluded, and these results should be interpreted cautiously. Future multicenter, prospective studies, ideally stratified by inflammatory burden, are warranted to clarify the true impact of CE treatment on reproductive outcomes.

## Conclusion

In conclusion, in the general ART population, untreated CE patients showed comparable clinical pregnancy and live birth rates to non-CE patients, but a lower observed miscarriage risk in fresh embryo transfer cycles. No statistically significant improvement in reproductive outcomes was observed following antibiotic treatment, and cure of CE demonstrated only a nonsignificant trend toward benefit. Our findings suggest that CE has limited overall impact on ART outcomes, though its clinical relevance may be greater in high-risk populations. Further prospective studies are needed to determine the role of CE diagnosis and treatment in improving reproductive success.

## Data Availability

The original contributions presented in the study are included in the article/Supplementary Material, further inquiries can be directed to the corresponding authors.
