# Supplementary material for: Impact of chronic endometritis on assisted reproductive technology outcomes: a propensity score inverse probability weighting cohort study
Source: Front Cell Dev Biol. 2026 Jan 28;14:1749173. doi: 10.3389/fcell.2026.1749173 (PMC12910471; doi:10.3389/fcell.2026.1749173)
Supplement: Supplementary file 1 [file Table1.docx]

**Supplementary Table S1**: Baseline Characteristics of Women With Untreated vs. Treated CE Status Undergoing Fresh Cycles Before and After Propensity Score Inverse Probability Weighting (PS IPW).

| **Baseline Characteristic** | **Overall, n = 1075** | **Before weighting** | | **SMD** | **After weighting** | | **SMD** |
| --- | --- | --- | --- | --- | --- | --- | --- |
|  |  | **Untreated**  **n = 534** | **Treated**  **n = 541** |  | **Untreated**  **n = 532.83** | **Treated**  **n = 543.06** |  |
| Age | 35.34 (4.86) | 35.32 (4.85) | 35.36 (4.88) | 0.009 | 35.36 (4.85) | 35.35 (4.83) | 0.003 |
| BMI (kg/m2) | 22.27 (3.14) | 22.31 (3.26) | 22.23 (3.01) | 0.025 | 22.25 (3.22) | 22.24 (3.03) | 0.005 |
| AMH (ng/mL) | 2.54 (2.03) | 2.46 (1.80) | 2.62 (2.23) | 0.081 | 2.51 (1.89) | 2.53 (2.04) | 0.008 |
| Gravidity | 1.54 (1.61) | 1.48 (1.51) | 1.60 (1.71) | 0.075 | 1.54 (1.56) | 1.54 (1.65) | 0.000 |
| Parity | 0.37 (0.62) | 0.39 (0.62) | 0.35 (0.62) | 0.062 | 0.37 (0.60) | 0.38 (0.64) | 0.006 |
| Infertility duration (y) | 5.39 (4.51) | 5.37 (4.51) | 5.41 (4.52) | 0.010 | 5.41 (4.57) | 5.40 (4.50) | 0.003 |
| Basal E2 (pg/mL) | 50.49 (93.63) | 51.37 (105.53) | 49.62 (80.25) | 0.019 | 49.77 (95.31) | 51.98 (82.51) | 0.024 |
| Basal FSH (mIU/mL) | 6.25 (3.11) | 6.21 (3.32) | 6.29 (2.88) | 0.025 | 6.28 (3.47) | 6.29 (2.86) | 0.002 |
| Basal LH (mIU/mL) | 3.64 (3.40) | 3.76 (3.61) | 3.52 (3.17) | 0.072 | 3.68 (3.12) | 3.91 (5.26) | 0.070 |
| Total Gn | 1982.29 (800.58) | 1974.46 (800.32) | 1990.01 (801.50) | 0.019 | 1984.14 (808.92) | 1982.02 (801.03) | 0.003 |
| Endometrial thickness | 11.34 (2.57) | 11.24 (2.50) | 11.43 (2.63) | 0.073 | 11.33 (2.55) | 11.33 (2.60) | 0.000 |
| No. of embryos transferred | 1.58 (0.49) | 1.57 (0.49) | 1.59 (0.49) | 0.037 | 1.58 (0.49) | 1.58 (0.49) | 0.006 |
| Fertilization method (%) |  |  |  | 0.092 |  |  | 0.002 |
| IVF | 832 (77.4) | 403 (75.5) | 429 (79.3) |  | 412.5 (77.4) | 420.8 (77.5) |  |
| ICSI | 243 (22.6) | 131 (24.5) | 112 (20.7) |  | 120.4 (22.6) | 122.2 (22.5) |  |
| Day of transfer (%) |  |  |  | 0.059 |  |  | 0.002 |
| Day 3 | 805 (74.9) | 393 (73.6) | 412 (76.2) |  | 400.2 (75.1) | 407.3 (75.0) |  |
| Day 5/6 | 270 (25.1) | 141 (26.4) | 129 (23.8) |  | 132.6 (24.9) | 135.7 (25.0) |  |
| Type of infertility (%) |  |  |  | 0.025 |  |  | 0.003 |
| Primary | 364 (33.9) | 184 (34.5) | 180 (33.3) |  | 180.3 (33.8) | 182.9 (33.7) |  |
| Secondary | 711 (66.1) | 350 (65.5) | 361 (66.7) |  | 352.6 (66.2) | 360.2 (66.3) |  |
| Infertility diagnosis (%) |  |  |  | 0.184 |  |  | 0.019 |
| Tubal factor | 732 (68.1) | 349 (65.4) | 383 (70.8) |  | 361.0 (67.7) | 366.6 (67.5) |  |
| Male factor | 167 (15.5) | 92 (17.2) | 75 (13.9) |  | 83.9 (15.7) | 88.5 (16.3) |  |
| Ovulatory | 99 (9.2) | 48 (9.0) | 51 (9.4) |  | 49.8 (9.3) | 50.3 (9.3) |  |
| Endometriosis | 18 (1.7) | 14 (2.6) | 4 (0.7) |  | 8.9 (1.7) | 8.6 (1.6) |  |
| Other | 59 (5.5) | 31 (5.8) | 28 (5.2) |  | 29.3 (5.5) | 29.1 (5.4) |  |
| Ovarian stimulation protocol, No. (%) | |  |  | 0.07 |  |  | 0.009 |
| Agonist | 612 (56.9) | 313 (58.6) | 299 (55.3) |  | 304.8 (57.2) | 309.3 (57.0) |  |
| Antagonist | 430 (40.0) | 206 (38.6) | 224 (41.4) |  | 211.8 (39.7) | 216.5 (39.9) |  |
| Mild Stimulation | 31 (2.9) | 14 (2.6) | 17 (3.1) |  | 15.2 (2.9) | 16.2 (3.0) |  |
| Natural cycles | 2 (0.2) | 1 (0.2) | 1 (0.2) |  | 1.0 (0.2) | 1.0 (0.2) |  |

Sample size after propensity score weighting is presented with decimal values because inverse probability weighting generates weighted pseudo-populations rather than integer counts. BMI, body mass index; AMH, anti-Müllerian hormone; E2, estradiol; FSH, follicle-stimulating hormone; LH, luteinizing hormone; Gn, gonadotropin; SMD, standardized mean difference; IVF, in vitro fertilization; ICSI, intracytoplasmic sperm injection.

**Supplementary Table S2**: Baseline Characteristics of Women with Persistent vs. Cured CE Status After Treatment Undergoing Fresh IVF Cycles Before and After PS IPW.

| **Baseline Characteristic** | **Overall, n = 284** | **Before weighting** | | **SMD** | **After weighting** | | **SMD** |
| --- | --- | --- | --- | --- | --- | --- | --- |
|  |  | **Cured CE**  **n = 127** | **Persistent CE**  **n = 157** |  | **Cured CE**  **n = 126.37** | **Persistent CE**  **n = 157.39** |  |
| Age | 35.01 (4.82) | 35.26 (5.09) | 34.81 (4.61) | 0.093 | 34.98 (4.95) | 35.08 (4.49) | 0.020 |
| BMI (kg/m2) | 22.36 (3.83) | 22.34 (4.77) | 22.39 (2.88) | 0.013 | 22.60 (5.59) | 22.37 (2.84) | 0.057 |
| AMH (ng/mL) | 2.82 (2.58) | 3.32 (3.23) | 2.42 (1.81) | 0.341 | 2.83 (2.55) | 2.92 (3.01) | 0.035 |
| Gravidity | 1.69 (1.84) | 1.57 (1.70) | 1.79 (1.94) | 0.122 | 1.63 (1.73) | 1.68 (1.84) | 0.028 |
| Parity | 0.37 (0.66) | 0.40 (0.69) | 0.34 (0.64) | 0.096 | 0.35 (0.63) | 0.35 (0.64) | 0.001 |
| Infertility duration (y) | 5.26 (4.30) | 5.83 (4.47) | 4.80 (4.12) | 0.242 | 5.31 (4.14) | 5.34 (4.60) | 0.006 |
| Basal E2 (pg/mL) | 49.99 (79.88) | 48.33 (76.78) | 51.34 (82.51) | 0.038 | 46.88 (72.09) | 48.78 (74.90) | 0.024 |
| Basal FSH (mIU/mL) | 5.98 (2.35) | 5.88 (1.92) | 6.06 (2.66) | 0.079 | 5.87 (1.81) | 5.97 (2.66) | 0.043 |
| Basal LH (mIU/mL) | 3.36 (1.95) | 3.57 (2.24) | 3.20 (1.66) | 0.186 | 3.33 (1.98) | 3.37 (1.84) | 0.020 |
| Total Gn | 2011.90 (789.11) | 1945.05 (810.90) | 2065.97 (769.39) | 0.153 | 2006.44 (809.55) | 2013.50 (767.58) | 0.009 |
| Endometrial thickness | 11.52 (2.47) | 11.45 (2.57) | 11.58 (2.40) | 0.055 | 11.47 (2.57) | 11.57 (2.33) | 0.038 |
| No. of embryos transferred | 1.59 (0.49) | 1.59 (0.49) | 1.59 (0.49) | 0.004 | 1.59 (0.49) | 1.60 (0.49) | 0.028 |
| Fertilization method (%) |  |  |  | 0.038 |  |  | 0.028 |
| IVF | 226 (79.6) | 100 (78.7) | 126 (80.3) |  | 98.8 (78.2) | 124.8 (79.3) |  |
| ICSI | 58 (20.4) | 27 (21.3) | 31 (19.7) |  | 27.6 (21.8) | 32.6 (20.7) |  |
| Day of transfer (%) |  |  |  | 0.044 |  |  | 0.010 |
| Day 3 | 211 (74.3) | 93 (73.2) | 118 (75.2) |  | 93.1 (73.7) | 116.6 (74.1) |  |
| Day 5/6 | 73 (25.7) | 34 (26.8) | 39 (24.8) |  | 33.3 (26.3) | 40.8 (25.9) |  |
| Type of infertility (%) |  |  |  | 0.002 |  |  | 0.006 |
| Primary | 96 (33.8) | 43 (33.9) | 53 (33.8) |  | 42.0 (33.2) | 52.7 (33.5) |  |
| Secondary | 188 (66.2) | 84 (66.1) | 104 (66.2) |  | 84.4 (66.8) | 104.7 (66.5) |  |
| Infertility diagnosis (%) |  |  |  | 0.249 |  |  | 0.051 |
| Tubal factor | 209 (73.6) | 96 (75.6) | 113 (72.0) |  | 91.4 (72.3) | 114.3 (72.6) |  |
| Male factor | 30 (10.6) | 9 (7.1) | 21 (13.4) |  | 16.0 (12.6) | 18.7 (11.9) |  |
| Ovulatory | 24 (8.5) | 11 (8.7) | 13 (8.3) |  | 9.2 (7.3) | 11.8 (7.5) |  |
| Endometriosis | 4 (1.4) | 3 (2.4) | 1 (0.6) |  | 1.7 (1.3) | 1.4 (0.9) |  |
| Other | 17 (6.0) | 8 (6.3) | 9 (5.7) |  | 8.2 (6.5) | 11.1 (7.1) |  |
| Ovarian stimulation protocol, No. (%) | |  |  | 0.173 |  |  | 0.092 |
| Agonist | 155 (54.6) | 67 (52.8) | 88 (56.1) |  | 70.8 (56.1) | 91.5 (58.2) |  |
| Antagonist | 120 (42.3) | 55 (43.3) | 65 (41.4) |  | 51.9 (41.1) | 61.7 (39.2) |  |
| Mild Stimulation | 8 (2.8) | 5 (3.9) | 3 (1.9) |  | 3.6 (2.9) | 3.6 (2.3) |  |
| Natural cycles | 1 (0.4) | 0 (0.0) | 1 (0.6) |  | 0.0 (0.0) | 0.6 (0.4) |  |

Data are presented as mean ± SD for continuous variables and as number (%) for categorical variables. Sample size after propensity score weighting is presented with decimal values because inverse probability weighting generates weighted pseudo-populations rather than integer counts.

**Supplementary Table S3**. Baseline Characteristics of Women With Non-CE and CE Status Undergoing FET Cycles Before and After PS IPW.

| Baseline Characteristic | Overall, n = 1014 | Before **weighting** | | SMD | After **weighting** | | SMD |
| --- | --- | --- | --- | --- | --- | --- | --- |
|  |  | **Non-CE**, n = 475 | **CE**, n = 539 |  | **Non-CE**, n = 474.97 | **CE**, n = 538.44 |  |
| Age | 34.90 (5.24) | 34.60 (5.15) | 35.17 (5.31) | 0.109 | 34.82 (5.15) | 34.87 (5.29) | 0.010 |
| BMI (kg/m2) | 22.29 (3.29) | 22.13 (3.08) | 22.43 (3.46) | 0.090 | 22.27 (3.14) | 22.29 (3.44) | 0.005 |
| AMH (ng/mL) | 3.63 (3.12) | 3.98 (3.57) | 3.32 (2.63) | 0.208 | 3.59 (3.25) | 3.59 (2.90) | 0.001 |
| Gravidity | 1.44 (1.58) | 1.44 (1.53) | 1.43 (1.62) | 0.007 | 1.40 (1.54) | 1.42 (1.62) | 0.013 |
| Parity | 0.29 (0.57) | 0.23 (0.48) | 0.35 (0.63) | 0.227 | 0.28 (0.54) | 0.29 (0.57) | 0.021 |
| Infertility duration (y) | 4.77 (4.04) | 4.56 (4.00) | 4.95 (4.07) | 0.097 | 4.79 (4.16) | 4.80 (3.96) | 0.004 |
| Endometrial thickness | 10.35 (2.15) | 10.10 (2.18) | 10.56 (2.10) | 0.216 | 10.35 (2.23) | 10.35 (2.10) | 0.001 |
| No. of embryos transferred | 1.32 (0.47) | 1.29 (0.45) | 1.35 (0.48) | 0.130 | 1.32 (0.47) | 1.32 (0.47) | 0.004 |
| Fertilization method (%) |  |  |  | 0.032 |  |  | 0.003 |
| IVF | 731 (72.1) | 346 (72.8) | 385 (71.4) |  | 341.3 (71.9) | 387.6 (72.0) |  |
| ICSI | 283 (27.9) | 129 (27.2) | 154 (28.6) |  | 133.7 (28.1) | 150.8 (28.0) |  |
| Type of infertility (%) |  |  |  | 0.129 |  |  | 0.008 |
| Primary | 252 (24.9) | 104 (21.9) | 148 (27.5) |  | 121.1 (25.5) | 135.3 (25.1) |  |
| Secondary | 762 (75.1) | 371 (78.1) | 391 (72.5) |  | 353.9 (74.5) | 403.1 (74.9) |  |
| Day of transfer (%) |  |  |  | 0.067 |  |  | 0.006 |
| Day 3 | 389 (38.4) | 174 (36.6) | 215 (39.9) |  | 185.5 (39.1) | 208.7 (38.8) |  |
| Day 5/6 | 625 (61.6) | 301 (63.4) | 324 (60.1) |  | 289.5 (60.9) | 329.7 (61.2) |  |
| Endometrial preparation, No. (%) | |  |  | 0.052 |  |  | 0.002 |
| Natural cycle | 236 (23.3) | 105 (22.1) | 131 (24.3) |  | 110.8 (23.3) | 125.3 (23.3) |  |
| Programmed cycle | 778 (76.7) | 370 (77.9) | 408 (75.7) |  | 364.1 (76.7) | 413.2 (76.7) |  |

Data are presented as mean ± SD for continuous variables and as number (%) for categorical variables. Sample size after propensity score weighting is presented with decimal values because inverse probability weighting generates weighted pseudo-populations rather than integer counts. BMI, body mass index; AMH, anti-Müllerian hormone; SMD, standardized mean difference; IVF, in vitro fertilization; ICSI, intracytoplasmic sperm injection; FET, frozen-thawed embryo transfer.

**Supplementary Table S4**. Baseline Characteristics of Women With Untreated vs Treated CE Status Undergoing FET Cycles Before and After PS IPW.

| Baseline Characteristic | Overall, n = 1059 | Before **weighting** | | SMD | After **weighting** | | SMD |
| --- | --- | --- | --- | --- | --- | --- | --- |
|  |  | **Untreated**  n = 539 | **Treated**  n = 520 |  | **Untreated**  n = 539.16 | **Treated**  n = 519.99 |  |
| Age | 35.07 (5.17) | 35.17 (5.31) | 34.96 (5.03) | 0.04 | 35.07 (5.30) | 35.08 (5.07) | 0.002 |
| BMI (kg/m2) | 22.37 (3.43) | 22.43 (3.46) | 22.30 (3.41) | 0.036 | 22.34 (3.47) | 22.35 (3.41) | 0.002 |
| AMH (ng/mL) | 3.44 (2.68) | 3.32 (2.63) | 3.56 (2.74) | 0.092 | 3.45 (2.78) | 3.45 (2.64) | 0.001 |
| Gravidity | 1.37 (1.59) | 1.43 (1.62) | 1.30 (1.55) | 0.081 | 1.37 (1.53) | 1.38 (1.72) | 0.006 |
| Parity | 0.31 (0.58) | 0.35 (0.63) | 0.27 (0.53) | 0.15 | 0.31 (0.59) | 0.31 (0.57) | 0.003 |
| Infertility duration (y) | 5.07 (4.18) | 4.95 (4.07) | 5.19 (4.28) | 0.057 | 5.06 (4.15) | 5.06 (4.18) | <0.001 |
| Endometrial thickness | 10.48 (2.18) | 10.56 (2.10) | 10.39 (2.26) | 0.082 | 10.47 (2.10) | 10.48 (2.30) | 0.003 |
| No. of embryos transferred | 1.34 (0.47) | 1.35 (0.48) | 1.33 (0.47) | 0.046 | 1.34 (0.47) | 1.34 (0.47) | 0.002 |
| Fertilization method (%) |  |  |  | 0.05 |  |  | <0.001 |
| IVF | 768 (72.5) | 385 (71.4) | 383 (73.7) |  | 391.1 (72.5) | 377.3 (72.6) |  |
| ICSI | 291 (27.5) | 154 (28.6) | 137 (26.3) |  | 148.0 (27.5) | 142.7 (27.4) |  |
| Type of infertility (%) |  |  |  | 0.005 |  |  | 0.003 |
| Primary | 290 (27.4) | 147 (27.3) | 143 (27.5 |  | 148.1 (27.5) | 143.6 (27.6) |  |
| Secondary | 769 (72.6) | 392 (72.7) | 377 (72.5) |  | 391.1 (72.5) | 376.4 (72.4) |  |
| Day of transfer (%) |  |  |  | 0.065 |  |  | <0.001 |
| Day 3 | 406 (38.3) | 215 (39.9) | 191 (36.7) |  | 206.9 (38.4) | 199.5 (38.4) |  |
| Day 5/6 | 653 (61.7) | 324 (60.1) | 329 (63.3) |  | 332.3 (61.6) | 320.5 (61.6) |  |
| Endometrial preparation, No. (%) | |  |  | 0.056 |  |  | 0.002 |
| Natural cycle | 270 (25.5) | 131 (24.3) | 139 (26.7) |  | 137.8 (25.6) | 132.5 (25.5) |  |
| Programmed cycle | 789 (74.5) | 408 (75.7) | 381 (73.3) |  | 401.4 (74.4) | 387.5 (74.5) |  |

Data are presented as mean ± SD for continuous variables and as number (%) for categorical variables. Sample size after propensity score weighting is presented with decimal values because inverse probability weighting generates weighted pseudo-populations rather than integer counts.

**Supplementary Table S5**. Baseline Characteristics of Women With Persistent vs Cured CE Status After Treatment Undergoing FET Cycles Before and After PS IPW.

| Baseline Characteristic | Overall, n = 282 | Before **weighting** | | SMD | After **weighting** | | SMD |
| --- | --- | --- | --- | --- | --- | --- | --- |
|  |  | **Cured CE**  n = 127 | **Persistent CE**  n = 155 |  | **Cured CE**  n = 128.59 | **Persistent CE**  n = 151.81 |  |
| Age | 35.08 (5.05) | 35.39 (5.63) | 34.83 (4.53) | 0.110 | 34.66 (5.63) | 35.08 (4.62) | 0.083 |
| BMI (kg/m2) | 22.27 (3.55) | 22.35 (3.99) | 22.20 (3.15) | 0.044 | 22.24 (3.70) | 22.08 (3.13) | 0.046 |
| AMH (ng/mL) | 3.70 (2.92) | 4.37 (3.51) | 3.16 (2.20) | 0.413 | 3.72 (2.95) | 3.53 (2.38) | 0.064 |
| Gravidity | 1.48 (1.73) | 1.32 (1.48) | 1.61 (1.90) | 0.166 | 1.38 (1.49) | 1.42 (1.76) | 0.024 |
| Parity | 0.28 (0.56) | 0.40 (0.65) | 0.19 (0.45) | 0.385 | 0.27 (0.54) | 0.23 (0.49) | 0.062 |
| Infertility duration (y) | 4.74 (3.84) | 4.32 (3.46) | 5.09 (4.10) | 0.202 | 4.66 (3.80) | 4.77 (3.77) | 0.028 |
| Endometrial thickness | 10.23 (2.38) | 10.29 (2.38) | 10.18 (2.39) | 0.047 | 10.24 (2.37) | 10.20 (2.31) | 0.016 |
| No. of embryos transferred | 1.30 (0.46) | 1.31 (0.46) | 1.30 (0.46) | 0.008 | 1.31 (0.46) | 1.32 (0.47) | 0.029 |
| Fertilization method (%) |  |  |  | 0.213 |  |  | 0.029 |
| IVF | 214 (75.9) | 90 (70.9) | 124 (80.0) |  | 98.3 (76.5) | 114.2 (75.2) |  |
| ICSI | 68 (24.1) | 37 (29.1) | 31 (20.0) |  | 30.3 (23.5) | 37.6 (24.8) |  |
| Type of infertility (%) |  |  |  | 0.048 |  |  | 0.080 |
| Primary | 81 (28.7) | 38 (29.9) | 43 (27.7) |  | 35.1 (27.3) | 47.0 (31.0) |  |
| Secondary | 201 (71.3) | 89 (70.1) | 112 (72.3) |  | 93.4 (72.7) | 104.8 (69.0) |  |
| Day of transfer (%) |  |  |  | 0.130 |  |  | 0.013 |
| Day 3 | 97 (34.4) | 48 (37.8) | 49 (31.6) |  | 46.2 (35.9) | 55.5 (36.6) |  |
| Day 5/6 | 185 (65.6) | 79 (62.2) | 106 (68.4) |  | 82.4 (64.1) | 96.3 (63.4) |  |
| Endometrial preparation, No. (%) | |  |  | 0.072 |  |  | 0.039 |
| Natural cycle | 75 (26.6) | 36 (28.3) | 39 (25.2) |  | 33.4 (26.0) | 42.1 (27.7) |  |
| Programmed cycle | 207 (73.4) | 91 (71.7) | 116 (74.8) |  | 95.2 (74.0) | 109.7 (72.3) |  |

Data are presented as mean ± SD for continuous variables and as number (%) for categorical variables. Sample size after propensity score weighting is presented with decimal values because inverse probability weighting generates weighted pseudo-populations rather than integer counts.

**Supplementary Table S6**. Interaction Effects Between CE Status and Baseline Characteristics on Pregnancy Outcomes in Fresh Embryo Transfer Cycles, Stratified by Treatment Group.

| Interaction | **Untreated group (CE [n=533.83] vs. Non-CE [n=432.35])** | | | **Treated (Persistent [n=157.39] vs. Cured CE [n=126.37])** | | |
| --- | --- | --- | --- | --- | --- | --- |
|  | clinical pregnancy | live birth | miscarriage | clinical pregnancy | live birth | miscarriage |
| CE: age | 1.00 (0.98 - 1.03) | 1.02 (0.99 - 1.06) | 0.97 (0.91 - 1.04) | 0.97 (0.93 - 1.02) | **0.95 (0.90 - 1.00)** | 1.07 (0.95 - 1.21) |
| CE: BMI | 1.00 (0.96 - 1.05) | 0.99 (0.94 - 1.05) | 1.03 (0.92 - 1.16) | 0.96 (0.90 - 1.02) | 0.97 (0.89 - 1.06) | 0.95 (0.79 - 1.13) |
| CE: AMH | 0.97 (0.92 - 1.03) | 0.96 (0.90 - 1.03) | 1.00 (0.83 - 1.21) | **1.07 (1.01 - 1.13)** | **1.08 (1.01 - 1.16)** | 0.98 (0.81 - 1.19) |
| CE: Gravidity | **1.11 (1.01 - 1.22)** | **1.14 (1.01 - 1.28)** | 1.08 (0.87 - 1.35) | 0.97 (0.84 - 1.10) | 0.90 (0.75 - 1.08) | 1.18 (0.88 - 1.58) |
| CE: Parity | 1.03 (0.77 - 1.39) | 1.06 (0.73 - 1.54) | 0.90 (0.44 - 1.83) | **1.51 (1.04 - 2.18)** | 1.53 (0.93 - 2.53) | 1.53 (0.68 - 3.46) |
| CE: years | 1.01 (0.97 - 1.05) | 1.01 (0.96 - 1.05) | 1.03 (0.94 - 1.12) | 1.02 (0.97 - 1.08) | 0.99 (0.93 - 1.07) | 1.09 (0.96 - 1.24) |
| CE: E2 | 1.00 (0.99 - 1.01) | 1.00 (1.00 - 1.00) | 0.99 (0.99 - 1.00) | **1.00 (0.99 - 1.00)** | 1.00 (0.99 - 1.00) | 0.99 (0.98 - 1.00) |
| CE: FSH | 1.00 (0.95 - 1.06) | 1.04 (0.97 - 1.12) | 0.98 (0.90 - 1.06) | 0.97 (0.86 - 1.10) | 0.96 (0.83 - 1.11) | 1.02 (0.70 - 1.49) |
| CE: LH | 1.00 (0.96 - 1.05) | 0.99 (0.94 - 1.03) | 1.10 (0.97 - 1.25) | 1.04 (0.92 - 1.17) | 1.05 (0.90 - 1.22) | 0.99 (0.69 - 1.42) |
| CE: total Gn | 1.00 (1.00 - 1.00) | 1.00 (1.00 - 1.00) | 1.00 (0.99 - 1.00) | 1.00 (1.00 - 1.00) | 1.00 (1.00 - 1.00) | 1.00 (1.00 - 1.00) |
| CE: ET | 0.98 (0.93 - 1.04) | 0.96 (0.90 - 1.02) | 1.05 (0.91 - 1.21) | 1.06 (0.97 - 1.15) | 1.08 (0.97 - 1.20) | 0.96 (0.75 - 1.24) |
| CE: No. of embryo transferred | 0.77 (0.58 - 1.01) | 0.92 (0.65 - 1.30) | **0.42 (0.19 - 0.91)** | 0.83 (0.53 - 1.30) | 1.06 (0.58 - 1.95) | 0.36 (0.10 - 1.27) |
| CE: ICSI | 0.93 (0.67 - 1.29) | 0.92 (0.62 - 1.38) | 0.91 (0.37 - 2.27) | 0.94 (0.57 - 1.55) | 0.84 (0.43 - 1.66) | 1.32 (0.28 - 6.29) |
| CE: Day 5/6 | 1.10 (0.84 - 1.43) | 0.87 (0.62 - 1.22) | **2.42 (1.01 - 5.81)** | 1.45 (0.94 - 2.22) | 1.26 (0.68 - 2.33) | 2.28 (0.64 - 8.19) |
| CE:Secondary infertility | 1.21 (0.92 - 1.60) | 1.19 (0.84 - 1.68) | 1.48 (0.66 - 3.34) | 0.86 (0.54 - 1.37) | 0.74 (0.40 - 1.38) | 1.68 (0.40 - 7.16) |
| CE: Male | 1.04 (0.71 - 1.53) | 0.98 (0.62 - 1.57) | 1.15 (0.37 - 3.63) | 0.76 (0.46 - 1.27) | 0.50 (0.23 - 1.10) | 3.22 (0.36 - 28.96) |
| CE: Ovulatory | 1.05 (0.57 - 1.95) | 0.79 (0.37 - 1.71) | 2.28 (0.56 - 9.26) | 0.56 (0.15 - 2.15) | 0.89 (0.13 - 5.95) | 0.40 (0.04 - 4.18) |
| CE: Endometriosis | 3.08 (0.77 - 12.37) | 4.62 (0.64 - 33.19) | 1.08 (0.07 - 16.64) | 1.13 (0.12 - 11.01) | 1.03 (0.10 - 10.12) | 1.60 (0.15 - 17.65) |
| CE: other | 1.37 (0.75 - 2.49) | 1.14 (0.51 - 2.56) | 2.38 (0.58 - 9.72) | 1.04 (0.37 - 2.91) | 2.80 (0.32 - 24.70) | 0.74 (0.09 - 5.76) |
| CE: Antagonist | 1.03 (0.76 - 1.39) | 1.01 (0.69 - 1.48) | 1.30 (0.61 - 2.77) | 0.59 (0.35 - 1.00) | **0.47 (0.24 - 0.94)** | 1.19 (0.30 - 4.70) |
| CE: Mild Stimulation | 1.12 (0.29 - 4.36) | 0.67 (0.12 - 3.84) | 2.60 (0.27 - 24.97) | 0.00 (0.00 - 0.00) | 0.00 (0.00 - 0.00) | 0.00 (0.00 - 0.00) |

Values are risk ratios (95% confidence intervals); BMI, body mass index; AMH, anti-Müllerian hormone; FSH, follicle-stimulating hormone; LH, luteinizing hormone; Gn, gonadotropin; ET, endometrial thickness; ICSI, intracytoplasmic sperm injection.

**Supplementary Table S7**. Interaction Effects Between CE Status and Baseline Characteristics on Pregnancy Outcomes in FET Cycles, Stratified by Treatment Group.

| Interaction | **Untreated group (CE [**n=538.44] **vs. Non-CE [**n=474.97]**)** | | | **Treated (Persistent [**n = 151.81] **vs. Cured CE [**n=128.59]**)** | | |
| --- | --- | --- | --- | --- | --- | --- |
|  | clinical pregnancy | live birth | miscarriage | clinical pregnancy | live birth | miscarriage |
| CE: age | 0.99 (0.97 - 1.01) | 1.00 (0.98 - 1.03) | 0.96 (0.90 - 1.02) | 0.97 (0.92 - 1.01) | 0.98 (0.92 - 1.03) | 0.92 (0.80 - 1.07) |
| CE: BMI | 0.99 (0.95 - 1.03) | 1.00 (0.95 - 1.05) | 0.98 (0.87 - 1.09) | 0.99 (0.92 - 1.06) | 0.97 (0.88 - 1.06) | 1.05 (0.90 - 1.23) |
| CE: AMH | 1.02 (0.99 - 1.05) | 1.00 (0.96 - 1.04) | 1.08 (0.96 - 1.21) | 1.01 (0.94 - 1.08) | 0.99 (0.89 - 1.09) | 1.10 (0.87 - 1.39) |
| CE: Gravidity | 0.96 (0.87 - 1.07) | 0.95 (0.84 - 1.09) | 1.04 (0.82 - 1.31) | 0.98 (0.80 - 1.20) | 0.95 (0.75 - 1.21) | 0.99 (0.55 - 1.79) |
| CE: Parity | 0.99 (0.75 - 1.31) | 0.93 (0.67 - 1.29) | 1.24 (0.54 - 2.84) | 1.42 (0.75 - 2.65) | 1.23 (0.57 - 2.64) | 2.37 (0.49 - 11.45) |
| CE: years | 0.99 (0.96 - 1.02) | 1.01 (0.97 - 1.05) | 0.95 (0.89 - 1.03) | 1.03 (0.97 - 1.10) | 1.06 (0.99 - 1.14) | 0.94 (0.77 - 1.15) |
| CE: ET | 0.98 (0.92 - 1.04) | 0.99 (0.92 - 1.06) | 0.93 (0.80 - 1.09) | 1.06 (0.96 - 1.17) | 1.10 (0.97 - 1.23) | 0.92 (0.67 - 1.25) |
| CE: No. of embryo transferred | 1.15 (0.85 - 1.54) | 1.20 (0.81 - 1.77) | 0.98 (0.47 - 2.07) | 1.63 (0.83 - 3.19) | 1.61 (0.73 - 3.54) | 1.75 (0.31 - 9.69) |
| CE: ICSI | 0.97 (0.74 - 1.28) | 0.96 (0.67 - 1.37) | 1.13 (0.52 - 2.45) | 1.11 (0.60 - 2.08) | 1.77 (0.85 - 3.67) | **0.08 (0.01 - 0.89)** |
| CE: Day 5/6 | 1.16 (0.76 - 1.78) | 1.20 (0.69 - 2.09) | 0.97 (0.40 - 2.35) | 0.93 (0.45 - 1.95) | 0.95 (0.39 - 2.31) | 0.87 (0.15 - 5.02) |
| CE: Secondary infertility | 0.84 (0.66 - 1.08) | 0.77 (0.56 - 1.06) | 1.24 (0.60 - 2.58) | 1.32 (0.80 - 2.20) | 1.23 (0.66 - 2.26) | 1.86 (0.36 - 9.72) |
| CE: Programmed cycle | 0.85 (0.62 - 1.16) | 0.77 (0.52 - 1.15) | 1.37 (0.55 - 3.43) | 1.19 (0.68 - 2.10) | 1.16 (0.59 - 2.28) | 1.36 (0.22 - 8.30) |

Values are risk ratios (95% confidence intervals).

**Supplementary Table S8.** Association between endometrial CD138⁺ plasma cell density and pregnancy outcomes in fresh embryo transfer and frozen embryo transfer cycles.

| Outcome | n/N (%) | Crude RR (95%) | Adjusted RR (95%) |
| --- | --- | --- | --- |
| Clinical pregnancy |  |  |  |
| 0 (negative) | 214/433 (49.4%) | Ref (1.0) | Ref (1.0) |
| 1-4 cells/HPF | 130/262 (49.6%) | 1.004 (0.853–1.182) | 1.018 (0.877–1.181)^a^ |
| ≥5 cells/HPF | 20/50 (40.0%) | 0.809 (0.561–1.167) | 0.749 (0.526–1.065) |
| live birth |  |  |  |
| 0 (negative) | 154/433 (35.6%) | Ref (1.0) | Ref (1.0) |
| 1-4 cells/HPF | 103/262 (39.3%) | 1.105 (0.901–1.357) | 1.127 (0.932–1.362) |
| ≥5 cells/HPF | 17/50 (34.0%) | 0.956 (0.629–1.453) | 0.882 (0.594–1.310) |
| miscarriage |  |  |  |
| 0 (negative) | 60/433 (13.9%) | Ref (1.0) | Ref (1.0) |
| 1-4 cells/HPF | 27/262 (10.3%) | 0.744 (0.480–1.153) | 0.780 (0.502–1.212) |
| ≥5 cells/HPF | 3/50 (6.0%) | 0.433 (0.140–1.336) | 0.441 (0.146–1.333) |
| FET |  |  |  |
| Clinical pregnancy |  |  |  |
| 0 (negative) | 242/477 (50.7%) | Ref (1.0) | Ref (1.0) |
| 1-4 cells/HPF | 120/254 (47.2%) | 0.931 (0.792–1.095) | 0.979 (0.844–1.136)^b^ |
| ≥5 cells/HPF | 23/38 (60.5%) | 1.193 (0.919–1.549) | 1.048 (0.830–1.323) |
| live birth |  |  |  |
| 0 (negative) | 182/477 (38.2%) | Ref (1.0) | Ref (1.0) |
| 1-4 cells/HPF | 94/254 (37.0%) | 0.970 (0.795–1.184) | 1.031 (0.857–1.240) |
| ≥5 cells/HPF | 20/38 (52.6%) | 1.379 (0.986–1.930) | 1.182 (0.854–1.637) |
| miscarriage |  |  |  |
| 0 (negative) | 58/477 (12.2%) | Ref (1.0) | Ref (1.0) |
| 1-4 cells/HPF | 25/254 (9.8%) | 0.809 (0.531–1.233) | 0.812 (0.531–1.243) |
| ≥5 cells/HPF | 2/38 (5.3%) | 0.433 (0.112–1.666) | 0.404 (0.106–1.535) |

HPF, high-power field; RR, relative risk; CI, confidence interval; FET, frozen embryo transfer. Plasma cell density was assessed by CD138 immunohistochemistry and, for analytic purposes, categorized as 0 (negative), 1–4 cells/HPF, and ≥5 cells/HPF.

RRs and 95% CIs were estimated using Poisson regression with a log link and patient-level cluster-robust standard errors to account for repeated embryo transfer cycles per patient. The reference group was 0 (negative). This sensitivity analysis was restricted to cycles performed during years in which quantitative CD138 reporting was available (2020–2023).

^a^ Adjusted models for fresh embryo transfer cycles included female age, BMI, AMH, gravidity, parity, duration of infertility, basal E2, FSH, LH, total gonadotropin dose, endometrial thickness, number of embryos transferred, fertilization method, day of embryo transfer, type of infertility, infertility diagnosis, and ovarian stimulation protocol.

^b^ Adjusted models for FET cycles included female age, BMI, AMH, gravidity, parity, duration of infertility, endometrial thickness, number of embryos transferred, fertilization method, day of embryo transfer, type of infertility, and endometrial preparation protocol.
